# Assessing the Impact of Glycine on *Elodea nuttallii* Growth, Photosynthesis, Antioxidant Activity, and Endogenous Hormones in a High-Temperature Environment

**DOI:** 10.3390/plants15172666

**Published:** 2026-08-31

**Authors:** Xinyue Shi, Jie Li, Liu Shao, Peimin He

**Affiliations:** 1 College of Oceanography and Ecological Science, Shanghai Ocean University, Shanghai 201306, China; 19816330148@139.com (X.S.); l1277172564@163.com (J.L.); pmhe@shou.edu.cn (P.H.); 2 Water Environment and Ecology Engineering Research Center of the Shanghai Institution of Higher Education, Shanghai Ocean University, Shanghai 201306, China

**Keywords:** antioxidant defense, high temperature stress, hormonal regulation, photosynthetic efficiency

## Abstract

High temperatures (HTs) severely constrain the growth and development of *Elodea nuttallii*, highlighting the need for effective mitigation strategies to maintain its ecological functions. This study investigated the effects of exogenous glycine (Gly) on *E. nuttallii* under high-temperature stress (HTS). Plants were assigned to three groups: (i) CK (no stress, 25 °C, no Gly), (ii) HT (HTS, 35 °C, no Gly), and (iii) HT+Gly (HTS, 35 °C, with Gly). HTS significantly reduced the plant height and fresh weight, chlorophyll fluorescence parameters (*F_v_/F_m_*, YII, and NPQ), photosynthetic pigments (Chl-*a*, Chl-*b*, and *Car*), CAT activity, and 6-BA content, while increasing the ABA, IAA, and GA levels. Exogenous Gly application substantially alleviated these adverse effects. Compared with the HT group, Gly increased *F_v_/F_m_*, YII, Chl-*a*, and Chl-*b* by 16.67%, 17.34%, 86.72%, and 72.22%, respectively, on day 4. Gly also strengthened antioxidant defense by increasing APX activity to as much as 2.38-fold that of the HT group and reducing H_2_O_2_ content by 29.03% on day 6. In addition, Gly restored a more balanced phytohormone profile. These findings indicate that exogenous Gly can substantially improve the HT tolerance of *E. nuttallii* and may provide a practical approach for enhancing macrophyte resilience under high-temperature conditions.

## 1. Introduction

Global warming is an ongoing challenge, and the average number of high-temperature (HT) days in China has increased significantly since the 1960s [1]. Rising temperatures pose a serious threat to the stability of aquatic ecosystems [2]. Elevated water temperatures can promote water acidification, alter the nutrient dynamics, and shift the community composition in aquatic environments [3,4,5].

Submerged macrophytes are key primary producers in aquatic ecosystems, providing habitat, nutrients, and energy that support higher trophic levels [6]. *E. nuttallii*, for example, is widely cultivated in Chinese mitten crab (*Eriocheir sinensis* H. Milne-Edwards) aquaculture because of its rapid reproduction and high biomass yield; however, it is highly sensitive to elevated temperatures [7]. Submerged macrophyte communities are increasingly threatened by high-temperature stress (HTS) [8]. Previous studies have shown that the growth of *E. nuttallii* is inhibited at water temperatures above 30 °C, with temperatures above 38 °C reducing the total dry weight by up to 1.47-fold relative to controls [9]. HTS can also disrupt antioxidant systems, alter endogenous hormone levels, and induce oxidative membrane damage in submerged macrophytes [10,11]. Collectively, these effects increase the ecological stress on *E. nuttallii* and threaten the stability of the aquatic ecosystems it supports [12]. Therefore, improving the HT tolerance of *E. nuttallii* is of considerable ecological importance.

Several strategies have been developed to mitigate the adverse effects of HTS on plants, including microbial-based approaches, genetic engineering, and the application of exogenous amino acids [13,14,15]. Microbial approaches can provide a sustainable means of enhancing thermal resilience, but they may raise ecological concerns, such as the displacement of native microbial communities and potential biosafety risks [16,17]. Although genetic engineering is advancing rapidly, its broader application remains constrained by technical challenges, including limitations in gene editing (e.g., protospacer adjacent motif specificity and the regeneration of recalcitrant species) and physical barriers such as the plant cell wall and organellar membranes, which can impede the efficient delivery of genetic material [18,19]. By contrast, exogenous amino acids offer a practical alternative because of their cost-effectiveness, ease of application, and relatively small environmental footprint, making them particularly attractive for improving the HT tolerance in complex aquatic ecosystems.

Glycine (Gly), a common amino acid and essential biological molecule, has been implicated in stress mitigation through the regulation of nitrogen metabolism, antioxidant defenses, and photosynthetic processes [20,21]. For example, Gly alleviated HTS in tomato seedlings by enhancing the antioxidant capacity, modulating the metabolic pathways, and providing osmotic protection [22]. Cao et al. [23] also reported that Gly improved the cold tolerance in rice by regulating the nitrogen uptake, physiological processes, and photosynthetic activity. To determine whether Gly can alleviate HTS in *E. nuttallii*, we evaluated the plant growth, photosynthetic performance, antioxidant defense, and endogenous phytohormones. Principal component analysis (PCA) was also used to integrate multiple indicators and provide a comprehensive assessment of the plant responses to HTS. These findings provide new insights into the potential use of Gly in the ecological restoration of submerged macrophytes.

## 2. Results

### 2.1. Effects on Plant Height and Fresh Weight

**Figure 1:**
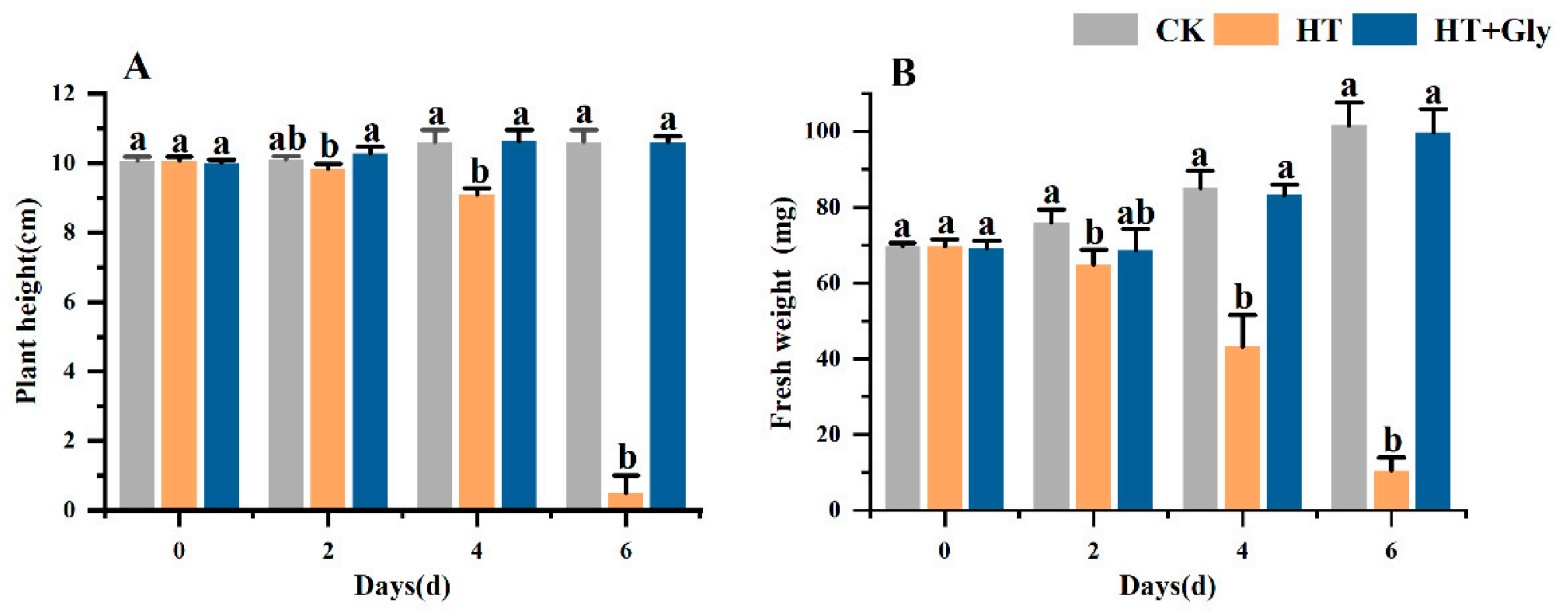
Effects of Gly application on the plant height (**A**) and fresh weight (**B**) of *E. nuttallii*. CK: no stress, 25 °C, no Gly treatment; HT: HT stress, 35 °C, no Gly treatment; HT+Gly: HT stress, 35 °C, with Gly treatment. Data labelled with different lowercase letters are significantly different at *p* < 0.05 level.

Under HTS, the plant height and fresh weight progressively declined relative to the CK group (Figure 1). On day 4, the plant height and fresh weight in the HT group were 14.15% (Figure 1A) and 49.30% (Figure 1B) lower, respectively, than those in the CK group, and both parameters had declined by more than 90% by day 6 (*p* < 0.05). In contrast, the plant height and fresh weight in the Gly group did not differ significantly from those in the CK group throughout the experiment, indicating that Gly application largely maintained normal growth under HTS.

### 2.2. Effects on Chlorophyll Fluorescence

**Figure 2:**
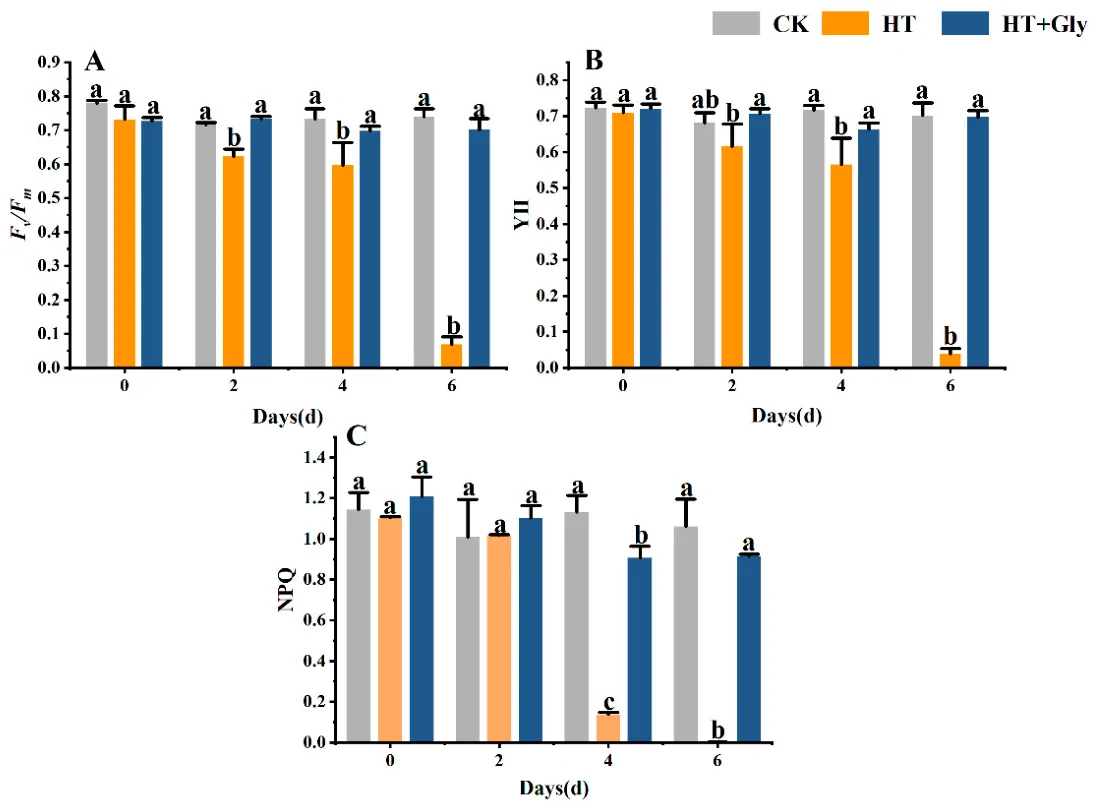
Effects of Gly application on the *F_v_/F_m_* (**A**), YII (**B**), and NPQ (**C**) of *E. nuttallii*. CK: no stress, 25 °C, no Gly treatment; HT: HT stress, 35 °C, no Gly treatment; HT+Gly: HT stress, 35 °C, with Gly treatment. Data labelled with different lowercase letters are significantly different at *p* < 0.05 level.

Changes in chlorophyll fluorescence parameters are shown in Figure 2. Relative to the CK group, *F_v_/F_m_*, YII, and NPQ were significantly reduced in the HT group from day 4 onward (*p* < 0.05). By day 6, *F_v_/F_m_* and YII (Figure 2A,B) had decreased by 90.68% and 94.58%, respectively. NPQ also declined rapidly from day 4 (Figure 2C). In contrast, *F_v_/F_m_*, YII, and NPQ in the Gly group did not differ significantly from those in the CK group throughout the experiment (*p* > 0.05; Figure 2).

### 2.3. Effects on Photosynthetic Pigments

**Figure 3:**
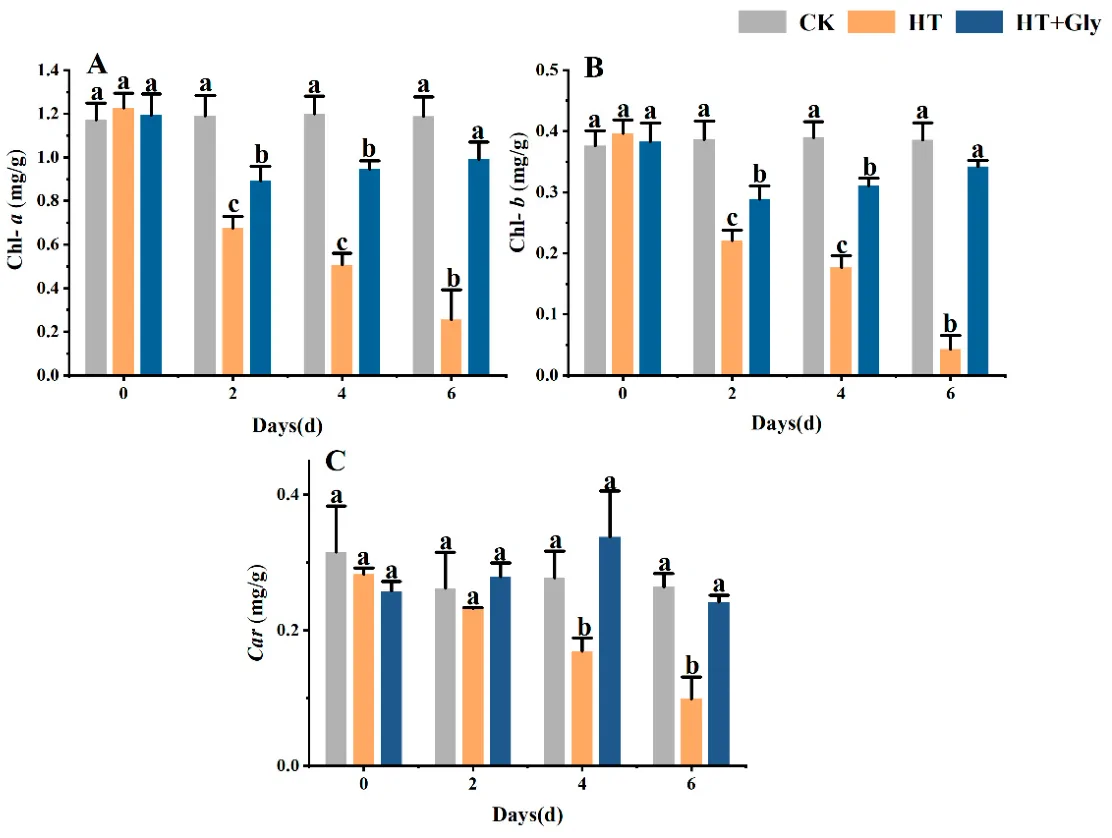
Effects of Gly application on the Chl-*a* (**A**), Chl-*b* (**B**), and *Car* (**C**) of *E. nuttallii*. CK: no stress, 25 °C, no Gly treatment; HT: HT stress, 35 °C, no Gly treatment; HT+Gly: HT stress, 35 °C, with Gly treatment. Data labelled with different lowercase letters are significantly different at *p* < 0.05 level.

Changes in the photosynthetic pigment contents are shown in Figure 3. In the HT group, the Chl-*a* and Chl-*b* contents progressively declined from day 2 onward relative to the CK group (Figure 3A,B) and were reduced by 78.15% and 89.47%, respectively, on day 6 (*p* < 0.05). The *Car* content decreased significantly from day 4 (Figure 3C), reaching a 62.86% reduction on day 6 (*p* < 0.05). Although the Chl-*a* and Chl-*b* contents in the Gly-treated group were lower than those in the CK group on days 2 and 4 (Figure 3A,B), they recovered to levels comparable to the CK group by day 6 (*p* > 0.05). The *Car* content in the Gly group remained relatively stable throughout the experiment and did not differ significantly from that in the CK group (*p* > 0.05; Figure 3C).

### 2.4. Effects on the Antioxidant System

**Figure 4:**
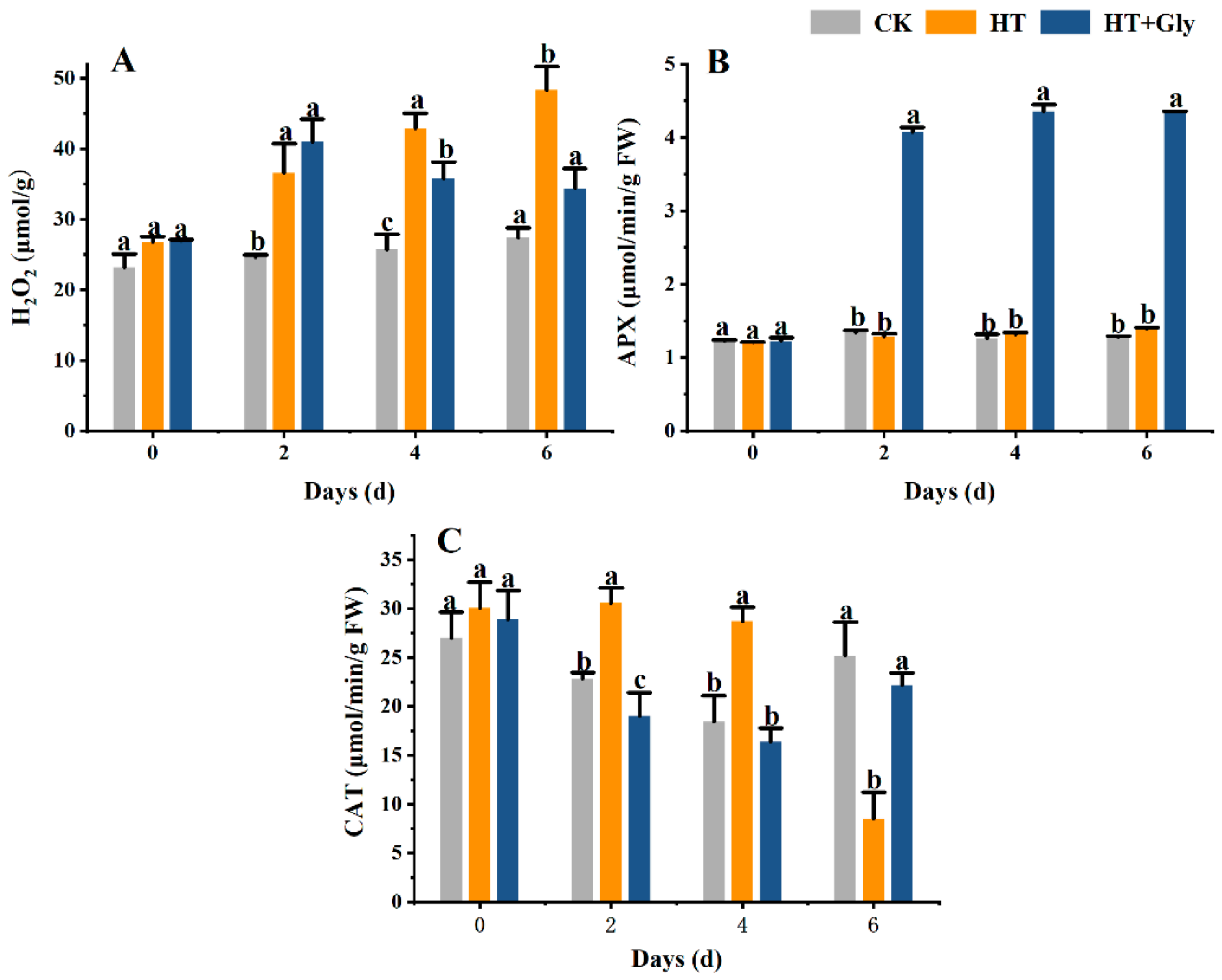
Effects of Gly application on the H_2_O_2_ (**A**), APX (**B**), and CAT (**C**) in *E. nuttallii*. CK: no stress, 25 °C, no Gly treatment; HT: HT stress, 35 °C, no Gly treatment; HT+Gly: HT stress, 35 °C, with Gly treatment. Data labelled with different lowercase letters are significantly different at *p* < 0.05 level.

As shown in Figure 4, HTS altered the antioxidant status of *E. nuttallii*. The H_2_O_2_ content in the HT group increased progressively (Figure 4A) and was 47.37%, 65.00%, and 76.19% higher than that in the CK group on days 2, 4, and 6, respectively (*p* < 0.05). The APX activity in the HT group did not differ significantly from that in the CK group and remained relatively stable throughout the experiment (*p* > 0.05; Figure 4B). The CAT activity in the HT group was higher than that in the CK group on days 2 and 4 (Figure 4C), but declined sharply on day 6, with a reduction of 66.27% (*p* < 0.05).

The Gly treatment mitigated these HT-induced changes in antioxidant status. Compared with the CK group, the H_2_O_2_ content in the Gly group was significantly higher by 63.16% and 35.00% on days 2 and 4, respectively (Figure 4A), but returned to the control level by day 6 (*p* > 0.05). The APX activity in the Gly group was 2.15-, 2.31-, and 2.31-fold that in the HT group on days 2, 4, and 6, respectively (*p* < 0.05; Figure 4B). The CAT activity in the Gly group did not differ significantly from that in the CK group (*p* > 0.05; Figure 4C), suggesting that Gly prevented the late-stage decline in CAT activity observed under HTS alone.

### 2.5. Effects on Endogenous Hormones

**Figure 5:**
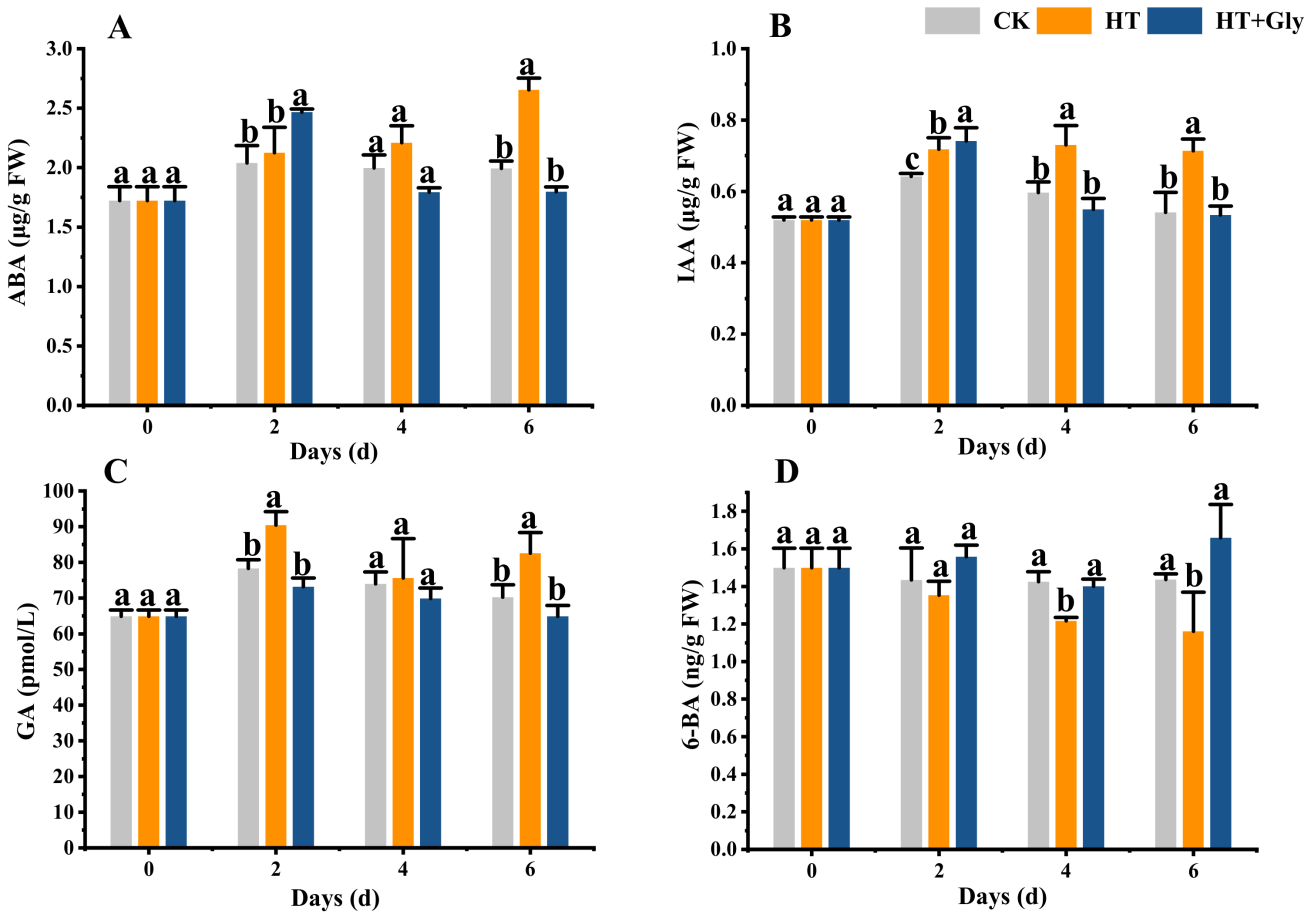
Effects of on the ABA (**A**), IAA (**B**), GA (**C**), and 6-BA (**D**) in *E. nuttallii*. CK: no stress, 25 °C, no Gly treatment; HT: HT stress, 35 °C, no Gly treatment; HT+Gly: HT stress, 35 °C, with Gly treatment. Data labelled with different lowercase letters are significantly different at *p* < 0.05 level.

Changes in the endogenous hormone levels are shown in Figure 5. In the HT group, the ABA content increased progressively (Figure 5A), rising by 40% after 2 days of HTS and reaching 2.65 μg/g by day 6 (*p* < 0.05). The IAA levels also showed an overall increasing trend (Figure 5B), with a significant peak of 0.72 μg/g on day 2 (*p* < 0.05). The GA levels increased by 15.46% and 17.54% on days 2 and 6, respectively (*p* < 0.05; Figure 5C). In contrast, the 6-BA content gradually declined (Figure 5D), with a significant reduction of 19.19% by day 6 (*p* < 0.05).

In the Gly group, the ABA and IAA levels increased transiently on day 2 by 16.98% and 21.01%, respectively (*p* < 0.05), but returned to control levels by day 4 (Figure 5A,B) and remained statistically similar to the CK group on days 4 and 6 (*p* > 0.05). The GA and 6-BA contents also remained comparable to those in the CK group at all time points (*p* > 0.05; Figure 5C,D).

### 2.6. PCA of the Mitigating Effects of Gly on E. nuttallii Under HTS

**Figure 6:**
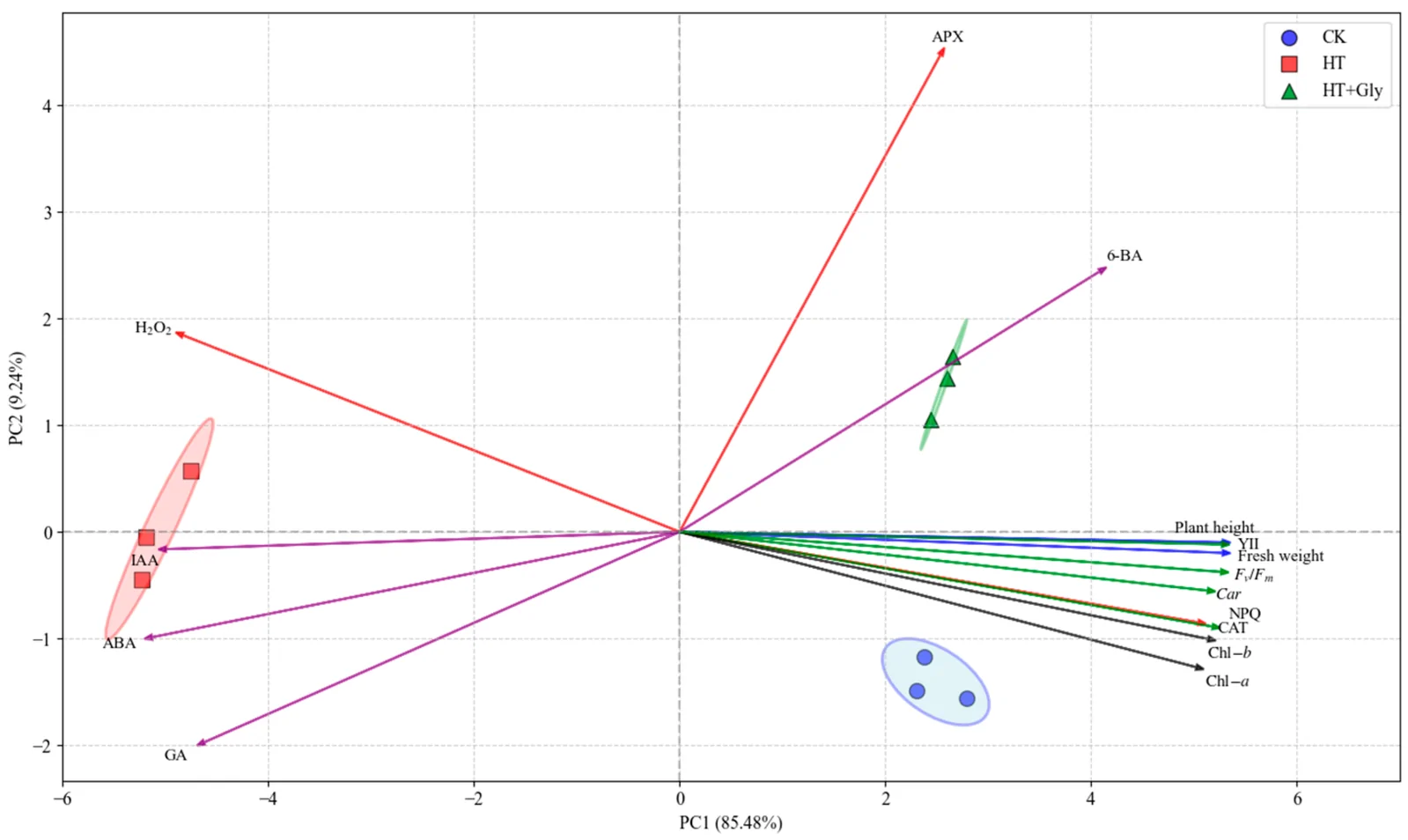
PCA of physiological and biochemical indicators of *E. nuttallii* under different treatments. The PCA displaying the distribution of samples from different treatments (CK: no stress, 25 °C, no Gly treatment; HT: HT stress, 35 °C, no Gly treatment; HT+Gly: HT stress, 35 °C, with Gly treatment) on day 6. The direction and length of the arrows indicate the strength and correlation of the variables with the principal components.

PCA was used to evaluate the effects of Gly on *E. nuttallii* under HTS. The first principal component (PC1) accounted for 85.48% of the total variance, while the second principal component (PC2) accounted for 9.24% (Figure 6). Samples from the HT group were clearly separated from those of the CK and Gly groups along PC1, indicating a marked shift in physiological status under HTS. In contrast, the Gly group clustered close to the CK group, suggesting that Gly largely alleviated HT-induced physiological changes and maintained an overall profile similar to that of the control. H_2_O_2_ showed a strong positive loading on PC1 and was closely associated with the HT group, consistent with enhanced oxidative stress under HTS. APX was more closely associated with the Gly group, indicating the activation of antioxidant defense following Gly application. In addition, the 6-BA, plant height, YII, fresh weight, *F_v_/F_m_*, *Car*, Chl-*a*, Chl-*b*, NPQ, and CAT were associated with the CK and Gly groups, reflecting better growth, photosynthetic performance, and antioxidant capacity. ABA, IAA, and GA were positioned closer to the HT samples, consistent with the HT-induced hormonal imbalance. Overall, the proximity of the Gly and CK clusters indicates that Gly effectively alleviated the HT-induced oxidative damage and hormonal disturbance in *E. nuttallii*.

## 3. Discussion

HTS imposes multiple interacting constraints on *E. nuttallii*, including oxidative damage, a hormonal imbalance, and the severe impairment of chloroplast function and PSII photochemistry. In this study, exogenous Gly enhanced the thermotolerance by strengthening the APX-based antioxidant defense, maintaining CAT activity, restoring a more favorable hormonal balance (ABA, IAA, GA, and 6-BA), and preserving the pigment content and PSII performance. Together, these responses suggest that a low-dose Gly application could serve as a simple, low-risk management approach to support the growth and ecological functions of cold-water submerged macrophytes during increasingly frequent aquatic heatwaves, for example, as a short-term thermal-protection measure during extreme events or as an aid to vegetation establishment in restoration projects. Nevertheless, because Gly is an organic nitrogen source that could stimulate microbial and algal growth, field application should first be evaluated in mesocosm and in situ experiments to ensure that its thermoprotective benefits do not compromise the water quality or community structure. The low concentration used in this study (0.01 mg/L), together with replacement every 2 days, may facilitate the sustained Gly uptake by *E. nuttallii* through foliar absorption and root-mediated transport. Submerged macrophytes can acquire amino acids through plasma-membrane-localized transporters (e.g., AAP and LHT families) and apoplastic pathways, while the frequent renewal of the external solution may maintain a concentration gradient that favors both passive diffusion and carrier-mediated uptake [24,25].

### 3.1. Alleviation of Oxidative Stress and Improvement in the Antioxidant Enzyme System by Gly Treatment

HTS disrupts redox homeostasis and promotes the excessive accumulation of reactive oxygen species (ROS), particularly H_2_O_2_, which can damage lipids, proteins, and chloroplast structures when concentrations exceed signaling thresholds [26,27]. In this study, *E. nuttallii* exposed to HTS showed a sustained increase in H_2_O_2_, consistent with previous observations in heat-stressed submerged macrophytes [8,11]. The transient increase in CAT activity during the early stage, followed by a sharp decline by day 6, suggests that peroxisomal H_2_O_2_ scavenging can compensate only temporarily before becoming impaired under prolonged oxidative stress [27,28].

In contrast, exogenous Gly markedly strengthened the antioxidant defense, as indicated by the 2.15–2.38-fold increase in APX activity and the subsequent return of H_2_O_2_ to near-control levels by day 6. APX is predominantly localized in chloroplasts and the cytosol and is considered a key enzyme for removing H_2_O_2_ generated by photosynthetic electron transport under heat and light stress [29]. The strong induction of APX, together with the maintenance of CAT activity in the Gly treatment, suggests that Gly not only enhances the overall antioxidant capacity but also preserves complementary chloroplast- and peroxisome-associated ROS-scavenging pathways. Thus, Gly may promote the APX-dominated detoxification of H_2_O_2_ near the chloroplast while preventing the late-stage decline in CAT activity observed in the HT group.

Maintaining efficient ROS-scavenging pathways may be critical for preventing system-wide redox disruption in submerged macrophytes. A similar Gly-induced enhancement in antioxidant systems has been reported in terrestrial species such as rice and tomato, where increased APX activity and reduced ROS accumulation were associated with improved performance under temperature stress [22,23]. Our results extend these findings to an aquatic macrophyte and suggest that the Gly-mediated enhancement in APX-centered antioxidant defense contributes to HT tolerance across contrasting plant growth forms. The elevated APX activity observed after Gly treatment may also partly reflect the increased de novo synthesis of antioxidant enzymes, because Gly can provide nitrogen and carbon skeletons for protein biosynthesis. However, the total soluble protein content was not measured in the present study, and this possibility should be examined further using proteomic or immunoblotting approaches [30].

### 3.2. Rebalancing of Multiple Hormonal Pathways Disrupted by HTS by Gly Application in E. nuttallii

#### 3.2.1. Mitigation of ABA Overaccumulation Under HTS by Gly Application in *E. nuttallii*

ABA, a key integrator of abiotic stress signals, is rapidly induced under HTS and activates antioxidant and other protective genes [31,32]. The progressive increase in ABA in the HT treatment is consistent with previous reports in submerged plants under stress conditions [33] and may contribute to the transient stimulation of antioxidant enzymes. However, persistently high ABA levels can suppress cell expansion and photosynthesis [34,35], consistent with the strong reductions in plant height and fresh weight observed in the present study.

The Gly treatment allowed a modest early increase in ABA but prevented its continued accumulation on days 4 and 6. This pattern suggests that Gly does not simply suppress ABA signaling; rather, it modulates ABA dynamics by permitting an initial stress response while avoiding prolonged ABA accumulation and the associated inhibition of growth. Such regulation may help *E. nuttallii* maintain a more favorable balance between defense activation and growth under HTS.

#### 3.2.2. Restoration of 6-BA Homeostasis Under HTS by Gly Application in *E. nuttallii*

Cytokinins (CKs) generally contribute to thermotolerance by promoting chloroplast development, enhancing ROS scavenging, and regulating heat-shock responses [36,37,38]. The marked decline in 6-BA under HTS therefore suggests that CK biosynthesis and/or stability is highly temperature-sensitive in *E. nuttallii* and may contribute to the observed instability of chlorophyll pigments and antioxidant systems. Similar decreases in CK content have been reported in *Sophora davidii* under low-phosphorus stress [39].

In the Gly treatment, 6-BA levels remained close to those of the control throughout the experiment despite exposure to HTS. This maintenance of CK homeostasis was consistent with the preservation of photosynthetic pigments and CAT activity. These results suggest that Gly may indirectly protect the chloroplast structure and function by stabilizing CK-dependent signaling pathways that are otherwise disrupted at high temperature. Maintaining CK homeostasis may therefore represent an important component of Gly-mediated thermotolerance in *E. nuttallii*.

#### 3.2.3. Normalization of Thermomorphogenic-like Increases in IAA and GA by Gly Application Under HTS in *E. nuttallii*

Under non-stress conditions, elevated temperatures can activate IAA and brassinosteroid (BR) biosynthesis through the phyB-PIF4/5/7 signaling axis, promoting stem elongation (thermomorphogenesis) as an adaptive response to warmer environments [40]. Heat stress, however, often suppresses GA synthesis to restrict vegetative growth and prioritize resource allocation to defense [41,42]. In the present study, *E. nuttallii* showed increased levels of both IAA and GA under HTS, deviating from the expected suppression of GA. This pattern suggests a growth-biased hormonal state and an imbalance in resource allocation between growth and defense, similar to responses reported in some submerged macrophytes [43,44]. In aquatic environments, where mechanical constraints are relatively low, early stem or shoot elongation may be adaptive because it can help plants access microhabitats with more favorable light or temperature conditions. However, when elongation occurs together with severe PSII impairment and substantial pigment loss, this growth-biased response may become maladaptive. A reduced photosynthetic capacity limits the energy and assimilates available to sustain elongation, potentially turning an initially beneficial strategy into a metabolic burden.

Gly treatment prevented the sustained accumulation of IAA and GA and restored both hormones to levels comparable to those of the control. In the Gly group, the transient increase in ABA on day 2 (16.98% above the control) subsided by day 4 and remained at control levels thereafter. This response further indicates that Gly modulates the temporal dynamics of stress-related hormones rather than simply suppressing stress signaling. Together with the normalization of 6-BA, these changes point to a broader rebalancing of the hormonal network rather than isolated effects on individual hormones. The PCA results support this interpretation: ABA, IAA, and GA were closely associated with HT samples, whereas 6-BA and growth- and photosynthesis-related traits were associated with the Gly and control groups. Thus, Gly appears to shift the hormonal state of *E. nuttallii* away from a stress-driven, growth-biased response toward a more coordinated balance between growth and defense, consistent with the recovery of photosynthetic performance.

### 3.3. Preservation of Chloroplast Integrity and Protection of PSII Photochemistry by Gly Application Under HTS in E. nuttallii

Under HTS alone, *E. nuttallii* showed progressive declines in Chl-*a*, Chl-*b*, and *Car*, accompanied by a near-complete loss of *F_v_/F_m_*, YII, and NPQ by day 6. These changes are consistent with the severe disruption of thylakoid membranes, the accelerated pigment degradation under oxidative stress, and the substantial impairment of the PSII reaction center. The concurrent declines in the PSII photochemical efficiency and NPQ indicate that both energy conversion and thermal dissipation were impaired, resulting in disrupted energy partitioning within the photosynthetic electron transport chain.

The Gly treatment substantially alleviated these effects. Although the Chl-*a* and Chl-*b* contents decreased slightly at early time points, both recovered to control levels by day 6, while *F_v_/F_m_*, YII, and NPQ remained comparable to those of the control throughout the experiment. Because Gly is both a precursor for chlorophyll biosynthesis and an organic nitrogen source, exogenous Gly may support pigment synthesis and the repair of PSII core proteins under HTS [21]. In addition, the enhanced APX activity and the restoration of redox balance may reduce the oxidative damage to the D1 protein and thylakoid lipids, thereby contributing to PSII stability [45,46].

Similar protective effects of Gly on the photosynthetic apparatus have been reported in rice and tomato seedlings [22,23]. Our findings extend these observations to a submerged macrophyte and indicate that the Gly-mediated protection of chloroplast function and PSII photochemistry is closely linked to its roles in nitrogen metabolism and ROS detoxification.

## 4. Materials and Methods

### 4.1. Experimental Materials

Gly (99% purity) and Hoagland nutrient solution were purchased from Shanghai Macklin Biochemical Technology Co., Ltd. (Shanghai, China).

The *E. nuttallii* specimens used in this study were cultivated, accessions obtained from an aquatic plant company (Shanghai Yuetian Biotechnology Co., Ltd., Shanghai, China). The selected *E. nuttallii* specimens were cleaned with tap water to remove surface attachments, and then pre-cultured in laboratory aquariums (60.0 cm length, 35.0 cm width, 35.0 cm height) at 25.0 ± 0.5 °C under a 16 h:8 h light: dark cycle. The photosynthetically active radiation (PAR) intensity was maintained at 140.5 μmol/m^2^/s. The sediment used in this experiment was collected from the campus river of Shanghai Ocean University (30°52′57.904″ N, 121°53′49.690″ E).

### 4.2. Experimental Design

A preliminary experiment was conducted to determine the optimal Gly concentration and replacement interval. Four Gly concentrations (0, 0.01, 0.1, and 1 mg/L) and two replacement intervals (replacement of the culture solution with fresh solution containing 0.01 mg/L Gly every 2 or 4 days) were evaluated. Based on the results (Figure S1), a Gly concentration of 0.01 mg/L with solution replacement every 2 days was selected for the subsequent experiments.

The HTS experiment was divided into three groups: (i) CK group (no stress, 25 °C, without Gly treatment), (ii) HT group (HT stress, 35 °C, without Gly treatment), and (iii) Gly group (HT stress, 35 °C, with Gly treatment). *E. nuttallii* individuals (fresh weight, 70 ± 5 mg; plant height, 10.0 ± 0.2 cm) were transplanted into 5 L cultivation containers (15 cm in diameter × 20 cm in height), with 21 plants per container. Each container contained a 5 cm layer of air-dried sediment and 10% Hoagland nutrient solution, under a 16 h:8 h light: dark cycle with a PAR intensity of 140.5 μmol/m^2^/s.

In the CK group, plants were maintained at 25.0 ± 0.5 °C. In the HT group, plants were first exposed to 25.0 ± 0.5 °C, followed by stepwise 5 °C increases every 48 h until reaching 35 °C. In the Gly group, plants were subjected to the same temperature regime as the HT group, with the addition of Gly (final concentration of 0.01 mg/L in the water). Throughout the experiment, distilled water was replenished daily to maintain the initial water level. All groups included three parallel replicates. *E. nuttallii* samples were collected on days 0, 2, 4, and 6 for analysis.

### 4.3. Determination of Growth Indicators

After a predetermined growth period (7 d) under experimental conditions, the plant height (in mm) of each *E. nuttallii* plant in all groups was measured using a meter stick. The fresh weight (in mg) was determined with an electronic balance (0.1 mg resolution) after blotting off surface moisture.

### 4.4. Determination of Photosynthetic Fluorescence Parameters

Chlorophyll fluorescence parameters were measured using a pulse-amplitude-modulated (PAM) fluorescence monitoring system (Phyto-PAM, Walz, Effeltrich, Germany) to assess the photosynthetic ability. The parameters, including the maximum photochemical efficiency of photosystem II (*F_v_/F_m_*), effective PSII quantum yield (YII), and non-photochemical quenching (NPQ), were determined according to He et al. (2023) [47].

### 4.5. Determination of Photosynthetic Pigments

Following the method described by Nie et al. (2016) [48], Chl-*a*, Chl-*b*, and *Car* content were determined using 95% ethanol extraction and estimated as follows (Equations (1)–(3)):

(1) $$\mathrm{Chl}\text{-}a\;(\mathrm{mg}/L)\;=\;\left(13.95\;\times \;A_{665}\right)\;-\;\left(6.88\;\times \;A_{649}\right)$$

(2) $$\mathrm{Chl}\text{-}b\;(\mathrm{mg}/L)=\left(24.96\;\times \;A_{649}\right)-\left(7.32\;\times \;A_{665}\right)$$

(3) $$\mathrm{Car}\;(\mathrm{mg}/L)=\frac{1000A_{470}-2.05\mathrm{Chl}\text{-}a-114.8\mathrm{Chl}\text{-}b}{245}$$

In the formulae, *A*_470_, *A*_649_, and *A*_665_ represent the absorbance values at wavelengths of 470, 649, and 665 nm, respectively. These absorbance values were determined by a UV–Vis spectrophotometer. Chl-*a*, Chl-*b*, and *Car* are the concentrations of chlorophyll *a*, chlorophyll *b*, and carotenoid. Finally, the chlorophyl contents were calculated as milligrams per gram fresh weight.

### 4.6. Determination of Enzymatic and Nonenzymatic Antioxidant Activities

For enzyme extraction, 0.1 g of fresh plant leaf tissue was fully ground in liquid nitrogen and homogenized in 1 mL of pre-cooled 50 mM phosphate buffer (pH = 7.8). The homogenate was centrifuged at a designated speed for 20 min at 4 °C using a high-speed refrigerated centrifuge (Eppendorf 5810R, Eppendorf, Hamburg, Germany). Subsequently, the supernatants were collected and used to determine catalase (CAT) and ascorbate peroxidase (APX) activities with corresponding commercial assay kits (Yuanye Bio-Technology Co., Ltd., Shanghai, China), in accordance with the manufacturer’s protocols. All absorbance values were detected via a UV–Vis spectrophotometer (Shimadzu UV-2600, Shimadzu, Shimadzu, Kyoto, Japan) with stable optical resolution and photometric accuracy [49].

For hydrogen peroxide (H_2_O_2_) determination, 0.5 g of fresh leaf tissue was ground in liquid nitrogen and thoroughly mixed with 5 mL of pre-cooled acetone. The mixture was centrifuged at 12,000 rpm for 20 min at 4 °C using a high-speed refrigerated centrifuge (Eppendorf 5810R, Eppendorf, Hamburg, Germany). The resulting supernatant was used to determine H_2_O_2_ content with a corresponding commercial assay kit (Yuanye Bio-Technology Co., Ltd., Shanghai, China). Absorbance was measured using a UV–Vis spectrophotometer (Shimadzu UV-2600, Shimadzu, Kyoto, Japan) [50].

### 4.7. Determination of Endogenous Hormones

For endogenous hormone determination, 0.5 g of plant leaf sample was ground in liquid nitrogen and mixed with 0.1 mol/L potassium phosphate buffer (PBS, pH 7.0–7.4). The mixture was centrifuged at 12,000× *g* for 20 min at 4 °C, and then the supernatants were used to measure endogenous hormone content using the respective assay kits. Indole-3-acetic acid (IAA) and abscisic acid (ABA) ELISA kits were obtained from Bioroyee Biotechnology Co., Ltd. (Beijing, China). Gibberellic acid (GA) and 6-benzylaminopurine (6-BA) ELISA kits were obtained from Huabodeyi Biotechnology Co., Ltd. (Beijing, China).

### 4.8. Data Analysis

Data are presented as the mean ± SD of three replicates. Normality and homogeneity of variance were verified before statistical analysis. Differences among the 35 °C treatment groups and the 25 °C control group were assessed using one-way ANOVA followed by Tukey’s honestly significant difference (HSD) post hoc test for multiple comparisons. Statistical analyses were performed using SPSS version 16.0 (IBM SPSS Software, Chicago, IL, USA), and figures were generated using Origin 2021 (OriginLab, Northampton, MA, USA). Different lowercase letters indicate significant differences at *p* < 0.05 according to Tukey’s test.

## 5. Conclusions

This study showed that exogenous Gly substantially alleviated HT-induced damage in *E. nuttallii* by strengthening antioxidant defense and maintaining hormonal homeostasis. Gly markedly increased the APX activity and helped restore H_2_O_2_ to near-control levels, thereby reducing oxidative stress. It also moderated stress-related hormonal changes by limiting excessive ABA accumulation and maintaining IAA, GA, and 6-BA at levels comparable to those of the control. The periodic low-dose Gly application used in this study may therefore provide a feasible approach for mitigating heat stress in natural or managed aquatic systems, although field validation is still required.

Overall, these findings indicate that Gly can improve the resilience of submerged aquatic plants under HTS and provide a basis for the further evaluation of this approach in warming aquatic environments. Additional field trials are needed to determine the practical applicability of Gly in the ecological restoration of submerged macrophytes under HTS.

## Data Availability

The original contributions presented in this study are included in the article/Supplementary Material. Further inquiries can be directed to the corresponding author.

## Supplementary Materials

The following supporting information can be downloaded at: https://www.mdpi.com/article/10.3390/plants15172666/s1, Figure S1A: Effects of different Gly concentrations on the plant height (a) and fresh weight (b) of *E. nuttallii*. CK: 25 °C, no Gly; HT: 35 °C, no Gly; HT+0.01: 35 °C, 0.01 mg/L Gly treatment; HT+0.1: 35 °C, 0.1 mg/L Gly treatment; HT+1: 35 °C, 1 mg/L Gly treatment. Data labelled with different lowercase letters are significantly different at *p* < 0.05 level. Figure S1B. Effects of different frequencies of Gly dosing on the plant height (a) and fresh weight (b) of *E. nuttallii*. CK: 25 °C, no Gly; HT: 35 °C, no Gly; HT+T1: 35 °C, culture solution replaced with fresh solution containing 0.01 mg/L Gly every 2 days; HT+T2: 35 °C, culture solution replaced with fresh solution containing 0.01 mg/L Gly every 4 days. Data labelled with different lowercase letters are significantly different at *p* < 0.05 level. Figure S2. Comparison of growth parameters of *E. nuttallii* between HT and HT+Gly groups on day 6. A 10 cm white paper strip placed on the left serves as a scale bar for measuring plant length.

## Abbreviations

*F_v_/F_m_*	The maximum photochemical efficiency of the photosystem II
YII	Effective PSII quantum yield
NPQ	Non-photochemical quenching coefficient
Chl-*a*	Chlorophyll *a*
Chl-*b*	Chlorophyll *b*
*Car*	Carotenoids
APX	Ascorbate peroxidase
H_2_O_2_	Hydrogen peroxide
ABA	Abscisic acid
IAA	Indole-3-acetic acid
GA	Gibberellin
6-BA	6-benzylaminopurine
