# Supplementary material for: Assessing the Impact of Glycine on Elodea nuttallii Growth, Photosynthesis, Antioxidant Activity, and Endogenous Hormones in a High-Temperature Environment
Source: Plants (Basel). 2026 Aug 31;15(17):2666. doi: 10.3390/plants15172666 (PMC13567175; doi:10.3390/plants15172666)
Supplement: Supplementary file 1 [file plants-15-02666-s001.zip › plants-4513861-supplementary.pdf]

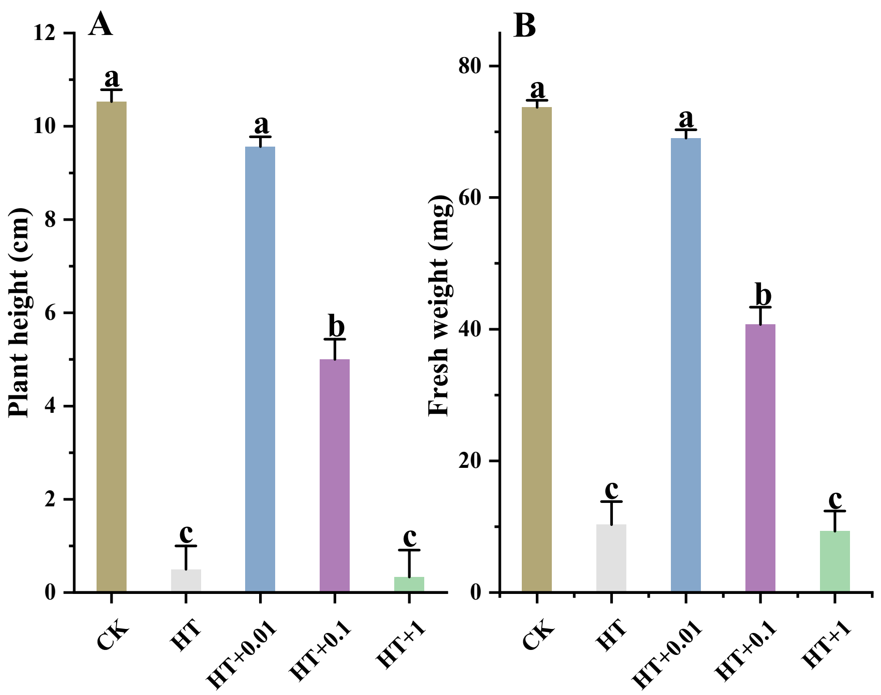

Figure S1A Effects of different Gly concentrations on the plant height (a) and fresh weight (b) of *E. nuttallii*. CK: 25°C, no Gly; HT: 35°C, no Gly; HT+0.01: 35°C, 0.01 mg/L Gly treatment; HT+0.1: 35°C, 0.1 mg/L Gly treatment; HT+1: 35°C, 1 mg/L Gly treatment. Data labelled with different lowercase letters are significantly different at $P < 0.05$ level.

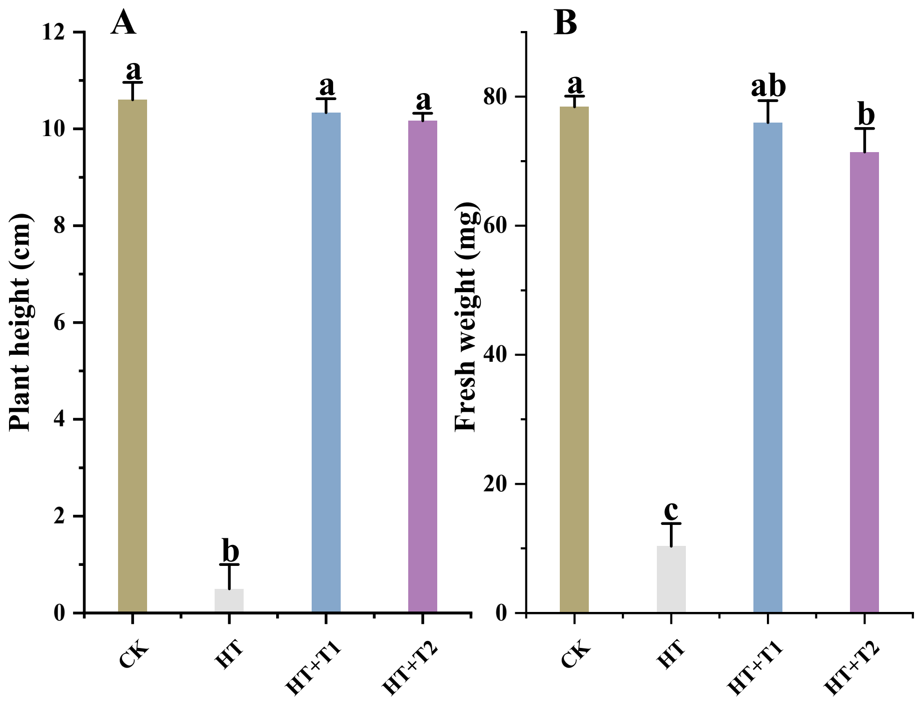

Figure S1B Effects of different frequencies of Gly dosing on the plant height (a) and fresh weight (b) of *E. nuttallii*. CK: 25°C, no Gly; HT: 35°C, no Gly; HT+T1: 35°C, culture solution replaced with fresh solution containing 0.01 mg/L Gly every 2 days; HT+T2: 35°C, culture solution replaced with fresh solution containing 0.01 mg/L Gly every 4 days. Data labelled with different lowercase letters are significantly different at $P < 0.05$ level.

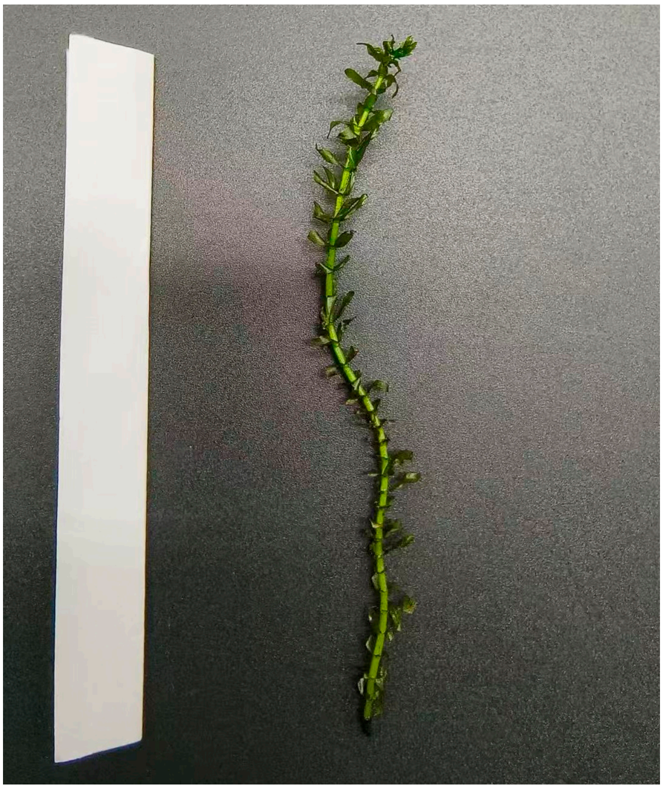

Figure S2 Comparison of growth parameters of *E. nuttallii* between HT and HT+Gly groups on day 6. A 10 cm white paper strip placed on the left serves as a scale bar for measuring plant length.
